# ‘I felt less alone knowing I could contribute to the forum’: psychological distress and use of an online infertility peer support forum

**DOI:** 10.1080/21642850.2021.1884556

**Published:** 2021-02-11

**Authors:** Siobhan Bernadette Laura O'Connell, Eden Noah Gelgoot, Paul Henry Grunberg, Joy Schinazi, Deborah Da Costa, Cindy-Lee Dennis, Zeev Rosberger, Phyllis Zelkowitz

**Affiliations:** a Sir Mortimer B. Davis Jewish General Hospital, Montreal, Canada; b Department of Psychiatry, McGill University, Montreal, Canada; c Department of Medicine, McGill University, Montreal, Canada; d Research Institute of the McGill University Health Centre, Montreal, Canada; e Lawrence S Bloomberg Faculty of Nursing, University of Toronto, Toronto, Canada; f Li Ka Shing Knowledge Institute, St Michael’s Hospital, Toronto, Canada; g Lady Davis Institute for Medical Research, Montreal, Canada; h Departments of Oncology, Psychology and Psychiatry, McGill University, Montreal, Canada

**Keywords:** Infertility, psychological distress, peer support, online forums, mixed-methods

## Abstract

**Background:**

Feelings of loss, shame and stigmatization, reduced quality of life, isolation and loneliness are common among men and women with infertility. Fertility patients may seek peer mentoring and support, specifically through the use of online forums, to fulfil their needs for shared experience and guidance through the fertility treatment process.

**Objective:**

To assess the use and benefits of an online fertility-related peer support forum through two research questions: (1) do socio-demographics, stress, and anxiety differ between posters on the forum, lurkers who read messages but did not post, and people who chose not to use it?; and (2) how did forum users describe their experiences?

**Design:**

A sample of 220 male and female fertility patients aged 23–54 years old (M = 35.51, SD = 4.94) were recruited at fertility clinics in Montreal and Toronto, Canada, to test a mobile application called ‘*Infotility*’. They answered questionnaires before and after being given access to *Infotility* for eight weeks. The peer support forum was accessible through the *Infotility* dashboard.

**Main Outcome Measures:**

Psychological distress was measured through the 4-item Perceived Stress Scale and the Generalized Anxiety Disorder 7-item Scale. Experiences using the forum were assessed through open-ended questions and in-depth interviews.

**Results:**

Participants with heightened psychological distress were more likely to become posters rather than lurkers or non-users and reported less distress after using the forum. Forum users appreciated the opportunity to share their experiences with others in similar situations.

**Conclusion:**

The forum reduced loneliness and allowed participants to learn new ways to manage stress. It was particularly beneficial for those with heightened psychological distress.

## Introduction

Infertility affects approximately 1 in 6 couples in Canada (Government of Canada, 2019) and is characterized by the failure to achieve a clinical pregnancy after twelve months or more of regular unprotected sexual intercourse or by a person’s inability to reproduce as an individual or with a partner (Zegers-Hochschild et al., 2017). Feelings of loss, shame and stigmatization, reduced quality of life, isolation and loneliness are common among men and women with infertility (Atwood & Dobkin, 1992; Greil, Slauson-Blevins, & McQuillan, 2010; Hinton, Kurinczuk, & Ziebland, 2010; Kiesswetter et al., 2019; Peterson, Newton, & Feingold, 2007). Fertility patients may seek peer mentoring and support, specifically through the use of online forums, to fulfil their needs for coping, shared experience, and guidance through the fertility treatment process (Read et al., 2014). Peer support, which refers to emotional, social and practical support provided by non-professionals with similar characteristics, offers an alternative mode of support than traditional forms of counselling (Dennis, 2003b; Grunberg, Dennis, Da Costa, & Zelkowitz, 2018).

The emergence of digital health has led to the development of online health-related peer support forums. Digital health refers to health care services and information provided by computers, mobile phones and satellite communications (Moss, Süle, & Kohl, 2019). Forums allow people to exchange experiences and receive empathetic responses from others who understand the challenges of infertility (Malik & Coulson, 2010a). A survey of fertility patients’ needs and preferences for online peer support found that most men (80.1%) and women (89.8%) expressed interest in online peer support, with higher stress levels related to greater interest. Respondents specifically valued online peer support that is monitored, accessible on a mobile platform and linked to relevant information (Grunberg et al., 2018).

Forums may foster feelings of community belonging by expanding social networks, serve as sources of information and social support, and help people cope with health-related struggles. Forum users appreciate the anonymity and find it easier to discuss their feelings online than in-person (Kummervold et al., 2002; Tanis, 2008). Fertility patients who have used forums were motivated by a desire to reduce isolation and communicate with people going through similar experiences. In using these forums, users felt more empowered and actively involved in the decision-making processes of their fertility treatment (Malik & Coulson, 2008; Sormunen, Karlgren, Aanesen, Fossum, & Westerbotn, 2020; van Uden-Kraan, Drossaert, Taal, Shaw, et al., 2008).

Potentially negative outcomes of using forums include waiting too long for responses (Himmel, Meyer, Kochen, & Michelmann, 2005), receiving inadequate information, or hearing personal stories such as pregnancy announcements, which may perpetuate negative emotions (Malik & Coulson, 2008, 2010b). These negative outcomes can be prevented or reduced in intensity if forums are monitored by trained peer supporters, who keep conversations on topic and respond appropriately if users exhibit worrisome or distressing behaviours.

The majority of users of fertility-related forums are women (Malik & Coulson, 2008; Sormunen et al., 2020). Forums may be particularly beneficial to those with mental health problems (Kummervold et al., 2002), which may be heightened among patients undergoing fertility treatment (Volgsten, Schmidt, Skoog Svanberg, Ekselius, & Sundström Poromaa, 2019). Furthermore, people who have used traditional mental health services (i.e. counselling) in the past may be more likely to turn to online health forums as a form of support, to supplement, but not replace traditional services (Kummervold et al., 2002).

While some people actively post and comment on forums (‘posters’), many people read forum threads but do not post anything themselves (‘lurkers’). Lurkers make up a large proportion of people who use forums, with studies showing that up to 60% of forum users are lurkers (Nonnecke & Preece, 2000; Walther & Boyd, 2002). Conceptualizations of ‘lurking’ have traditionally had negative connotations, defining lurkers as those who conceal themselves for the purpose of deceit or as free-riders who do not contribute in online communities (Kollock & Smith, 1996). More recently, research has challenged the negative connotations associated with lurking and has opted for more neutral ideas of what it means to be a lurker on the Internet (Nonnecke & Preece, 1999; Sun, Rau, & Ma, 2014). In this paper, the term ‘lurker’ is not considered to be negative. Instead, lurkers are considered to be silent but active users of online communities, who read discussions but do not post.

Lurkers and posters often have different motivations for using forums. Malik and Coulson (2011) found that among those who used online infertility support groups, lurkers were more likely to use them to receive hope and reassurance, whereas posters were more likely to use them for empathetic and emotional support. Research suggests that lurkers are shyer than posters, and often report that they do not feel the need to post, or do not feel comfortable sharing their own feelings or concerns (Preece, Nonnecke, & Andrews, 2004; Sun et al., 2014).

Some research on online forums for varying health conditions have found that lurkers and posters did not differ in age (Han, Hou, Kim, & Gustafson, 2014; Preece et al., 2004), gender, employment status (Preece et al., 2004; van Uden-Kraan, Drossaert, Taal, Shaw, et al., 2008), level of education (Han et al., 2014; Preece et al., 2004; van Uden-Kraan, Drossaert, Taal, Seydel, & van de Laar, 2008), or time since cancer diagnosis (Han et al., 2014). There have been inconsistent findings about differences in mental health between lurkers and posters (Han et al., 2014; van Uden-Kraan, Drossaert, Taal, Seydel, et al., 2008). Experiences and perceived benefits of using a forum might also depend on a user’s level of engagement. Research suggests that lurkers often report lower overall satisfaction with forums compared to posters, who feel a greater sense of membership and community (Malik & Coulson, 2011; Preece et al., 2004).

To the best of our knowledge, no study to date has compared posters, lurkers, and those who choose not to use fertility-related forums on measures of psychological distress and socio-demographic characteristics. Knowing who is most likely to be engaged with these forums will allow for a more nuanced understanding of how forums should be designed for fertility patients. In addition, there is a need to evaluate under which conditions and for whom forums are effective in reducing psychological distress (Eysenbach, Powell, Englesakis, Rizo, & Stern, 2004). The current study expands on existing literature by adopting a mixed-methods approach to understand who uses fertility-related forums, how they experience them, and how different levels of engagement may relate to perceptions of stress associated with current difficult life circumstances, as well as symptoms of anxiety.

A mobile application (app) called ‘*Infotility/Infotilité*’ was developed by our team of researchers and fertility health care professionals to provide fertility patients with a single-source of reliable information about the medical and psychosocial aspects of infertility. The app included a confidential peer support forum called ‘*Connect*’ that was monitored by trained peer supporters with experiences of infertility and its treatment.

The current paper addresses the following research questions through a mixed-methods approach: (1) do socio-demographic characteristics, stress, and anxiety differ between posters, lurkers, and people who chose not to use the *Connect* forum?; and (2) how did *Connect* users describe their experiences with using the forum and interacting with peer supporters?

## Materials and methods

### Participants

Fertility patients were recruited using a convenience sampling approach at four fertility clinics in Montreal and Toronto, Canada, from October 29, 2018, to December 17, 2018. Ethics approval was obtained from the institutional ethics review boards of each recruitment site. To be eligible for the study, participants had to be 18 years or older, identify as male or female, be in a relationship with someone of the opposite sex, be proficient in English or French, and have access to the Internet.

A total of 969 people (336 men, 633 women) were approached by recruiters, of whom 661 (220 men, 441 women) agreed to be screened for eligibility and 505 (164 men, 341 women) were eligible to participate. Of those who were eligible, 387 (124 men, 263 women) consented to be part of the study, 267 participants (77 men, 190 women) completed all of the intake questionnaires and were given access to the *Infotility* app, and 220 (50 men, 170 women) went on to use the app. Participants were sent three emails to encourage app use throughout the 8-week study period, as well as three automatic email reminders and up to five phone calls from their recruiter if they had not yet completed the follow-up questionnaires after using the app. Participants dropped out of the study or did not complete the follow-up questionnaires for a number of reasons, including being no longer interested, too busy, or mentally and/or physically distressed. The final study sample for this paper includes the 220 participants who visited at least one page on the app, not including the homepage. The sample was, on average, 35.51 years old (SD = 4.94, range 23–54). After eight weeks of using *Infotility*, 172 (78.2%) of the app users completed the follow-up questionnaires, and eleven participants engaged in interviews.

### Procedures

The *Infotility* app was presented to patients as a mobile application specifically designed for fertility patients, with doctor-approved information about the medical and psychological aspects of fertility, as well as access to an online peer support forum. Patients were asked if they would like to be part of the study evaluating the *Infotility* app, by using it for eight weeks and providing us feedback.

Individuals who were eligible and interested provided informed written consent. They were then sent a link by email to complete intake questionnaires including demographic questions and a number of standardized questionnaires measuring their perceived stress and anxiety symptoms, which took approximately 30 minutes to complete. After completing the intake questionnaires, participants were sent a link by email to access the *Infotility* app. Participants used *Infotility* as much or as little as they liked during the eight-week study period. App users could choose to access the *Connect* forum through a link on the *Infotility* homepage. Participants could use and read the articles on the informational side of the app without accessing the forum. The forum was monitored for ten hours a day, seven days a week by trained peer supporters.

After using *Infotility*, participants were asked to complete follow-up questionnaires about their perceived stress, anxiety symptoms and fertility treatment outcomes, in addition to questions about their experiences using the app and peer support forum. As participants used the app and completed the follow up questionnaires, they were invited to participate in individual, semi-structured, qualitative interviews over the phone about their experiences using *Infotility*. Interviews were audiotaped and transcribed into text by research assistants.

### Measures

#### Infotility app use

Google Analytics were used to measure the actual app use of each participant. Participants’ page views were tracked throughout their eight-week study period. Participants were considered to have used the *Connect* forum if they visited the forum at least once, as indicated by their page views. Based on whether or not they chose to access the *Connect* forum while using the app, the sample of 220 *Infotility* users was divided into three groups: (1) participants who posted on *Connect (‘posters’)*, (2) participants who read messages on *Connect* but did not post (‘lurkers’), and (3) participants who used the *Infotility* app but never accessed the *Connect* forum (‘non-users’).

#### Anxiety symptoms

The Generalized Anxiety Disorder 7-item Scale (GAD-7) is a screening tool for anxiety, widely used in health research. It assesses anxiety symptoms in the past two weeks and was administered at both intake and follow-up. The GAD-7 is made up of 7 Likert-scaled questions (range 0–3) and has excellent internal consistency, with a Cronbach’s alpha coefficient of 0.93 at intake and 0.92 at follow-up in the present study. A total score for the GAD-7 was generated by summing answers to each individual question (theoretical range 0–21), with a higher score representing higher levels of anxiety symptoms.

#### Perceived stress

The 4-item Perceived Stress Scale (PSS-4) was administered at intake and follow-up to measure the extent to which a participant felt as though the events in their life were stressful in the past month (Warttig, Forshaw, South, & White, 2013). The PSS-4 is widely used in health research and differs from the GAD-7 in that it measures perceptions of the stressful nature of current life circumstances, rather than an individual’s propensity to feel anxious in a variety of circumstances. The PSS-4 includes 4 Likert-scaled questions (range 0–4) and has satisfactory internal consistency, with a Cronbach’s alpha coefficient of 0.80 at intake and 0.79 at follow-up in the present study. A total score for the PSS-4 was generated by summing answers to each individual question (theoretical range 0–16), with a higher score representing higher levels of perceived stress.

#### Socio-demographic characteristics

Before using *Infotility*, participants were asked a number of socio-demographic questions including their sex, education level, annual household income, immigrant status, ethnicity, parity, type of infertility diagnosis, time in fertility treatment, and whether or not they had sought psychological counselling in the past (‘prior psychological counselling’). Education was measured as highest level of education completed (high school or less, CEGEP/trade/vocational, university degree, graduate degree). *Collège d’enseignement général et professionnel* (CEGEP) is a college preparatory or technical programme completed between high school and university in Quebec, Canada. Annual household income was categorized as less than 80,000 Canadian dollars (CAD); 80,000–119,999 CAD; 120,000–159,999 CAD; or 160,000 CAD and more. Immigrant status was dichotomized into ‘immigrant’ or ‘born in Canada.’ Ethnicity was dichotomized into ‘white’ or ‘non-white.’ Parity was dichotomized into ‘no children’ or ‘one or more children.’ Type of infertility diagnosis was categorized into female-factor, male-factor, mixed, or unexplained infertility. Time in fertility treatment was measured by the number of months since the participant had first visited a fertility specialist and was re-coded into an ordinal variable with four categories based on the inter-quartile range: less than 5 months, 5–12 months, 13–30 months, and 31 months or more. After using the app, participants were asked whether or not they had achieved a pregnancy throughout the 8-week study period.

#### Evaluation of peer supporters

The Peer Support Evaluation Inventory (PSEI) is a questionnaire developed by the *Infotility* team and adapted from a measure developed by Dennis (2003a) that measures participants’ experiences with the peer supporters on the *Connect* forum (see Appendix 1 for the list of items). The PSEI was administered after eight weeks of using the app. The PSEI consists of 16 Likert-scaled questions (range 1–4), which were summed to generate a total score (theoretical range 16–64). The higher the total score, the more positive experience the participant had with the forum. The PSEI total score has excellent internal consistency, with a Cronbach’s alpha coefficient of 0.91 from the present study. The PSEI also included one open-ended question for participants to expand on their experiences interacting with the peer supporters. Participants were instructed to only fill out the PSEI if they had interacted with the peers, and to skip it if they had not. Some people may have misinterpreted these instructions and skipped this questionnaire even if they had used the forum; for example, lurkers who did not interact directly with the peer supporters. In addition, some participants filled out the PSEI even if they had not used the forum. For the purposes of this paper, the PSEI scores of all those who were non-users of *Connect* were not included in analyses. Since participants were instructed to only fill out the PSEI if they had interacted with the peers, and since non-users were excluded from PSEI analyses, there was a smaller sample size (N = 42) when analyzing the PSEI scores in this paper.

#### Satisfaction with the *Connect* forum

Three open-ended questions were administered eight weeks after using the app: (1) ‘Please describe any fertility topics or any features that were not included on the app and that you would have liked to be included;’ (2) ‘Please tell us what you liked best about the app, and why;’ and (3) ‘Please tell us what you liked least about the app, and why.’ In addition, qualitative interviews were conducted to obtain a better understanding of participants’ experiences using the app, and whether any aspects could be improved upon. Interviews were semi-structured and lasted from 15 to 90 minutes. Participants were asked about what they liked best and least about the app, what they hoped to gain from using the app, their experiences using the forum and interacting with peer supporters, and how the app could be improved.

### Research design and analytic strategy

The *Infotility* project was designed to collect both quantitative and qualitative data, through self-report questionnaires, short-answer open-ended questions, and in-depth semi-structured qualitative interviews. A mixed-methods approach was used to explore the research questions and provide a nuanced explanation of the use and experiences of people on an online peer support forum designed for individuals going through fertility treatment. Quantitative analyses were used to analyze whether posters, lurkers and non-users differed in socio-demographic characteristics, psychological distress and PSEI ratings. Research indicates that data are only likely to be biased if more than 10% of data are missing (Bennett, 2001). At intake, the range of missing data for all variables used in the current study was between 0 and 4.5% and therefore listwise deletion was applied to handle missing data for quantitative analyses. Little’s MCAR test indicated that the data for our dependent variables of interest (GAD-7 and PSS-4) were missing completely at random at both intake and follow-up (chi-square  =  20.312, DF =  17, *p* = .259).

Chi-square tests were used to see if there were any significant differences among posters, lurkers and non-users in socio-demographic and fertility-related variables. ANOVAs with post-hoc Bonferroni tests were used to see if posters, lurkers and non-users differed significantly in their anxiety symptoms and perceived stress at intake. Two multinomial logistic regression models were performed to test if levels of perceived stress and anxiety symptoms at intake predicted whether the participant was a non-user, lurker or poster (with poster being the reference group), controlling for socio-demographic variables that were significant in bivariate analyses. The first regression model was performed with anxiety symptoms (GAD-7) as an independent variable, and the second model was performed with perceived stress (PSS-4) as an independent variable. GAD-7 and PSS-4 were highly correlated (*r*(207)  =  .689, *p*  <  .001), and therefore were not included in the same model. Paired sample t-tests were used to determine whether there were any significant changes in PSS-4 and GAD-7 scores from intake to follow-up for posters, lurkers and non-users. Repeated-measures ANCOVAs were run with *Connect* use as the between-subjects factor (non-users versus lurkers versus posters), time as the within-group factor (from intake to follow-up), and with an interaction term between group and time and controlling for all variables significant in bivariate analyses. An independent sample t-test was performed to determine whether posters and lurkers differed on their PSEI total score. The threshold for statistical significance was set at *p* < 0.05. However, due to the small sample size and exploratory nature of the current study, results with *p*-values approaching significance (*p* < 0.10) were also considered relevant and were discussed in the results (Thiese, Ronna, & Ott, 2016).

Three researchers (SO, EG, and JS) performed qualitative thematic analysis (Braun & Clarke, 2006, 2014) on the open-ended questions from the follow-up questionnaires and the qualitative interviews, to explore the experiences of participants who used the *Connect* forum, what aspects they appreciated, and what aspects could be improved upon. A data-driven approach was used, where transcripts were read iteratively without pre-conceived themes from previous literature. The 10th and 11th interviews did not reveal any new data that informed the thematic analysis. Recruitment of participants for interviews was terminated once interview themes reached saturation, which was achieved after 11 interviews (Guest, Bunce, & Johnson, 2006). Six themes pertaining to peer support and forum usage were identified by the three researchers through collaboration, and then the transcripts were re-read by each researcher to ensure the themes represented the data. All qualitative data that were not explicitly related to the *Connect* forum or experiences with peer supporters were excluded from thematic analysis. Researchers verified that each participant with qualitative data had actually used the *Connect* forum, ensuring that the participant was not referring to another form of peer support on the Internet. If a participant provided qualitative responses but had not used *Connect*, they were excluded from thematic analysis. All French quotations were translated into English for this paper. Thematic analysis was conducted using NVivo, and statistical analyses were conducted using IBM SPSS Statistics 19.

## Results

### Differences between posters, lurkers and non-users on the *Connect* forum

The 220 participants who used the *Infotility* app had an average of 37.44 (SD = 36.05) pageviews on the app. Forty of the 220 app users (18.18%) posted on the *Connect* forum, 66 (30.00%) lurked on the forum but did not post, and 114 (51.82%) only used the informational side of the app and did not access the *Connect* forum. Posters and lurkers were significantly more likely to be female (X^2^ (2, N = 220)  =  14.682, *p* = .001), to have been in fertility treatment longer (X^2^ (6, N = 212)  =  17.353, *p* = .008) and to have had prior psychological counselling (X^2^ (2, N = 218)  =  9.222, *p* = .010) compared to non-users. Posters and lurkers were also more likely to not have any children (X^2^ (2, N = 217)  =  5.493, *p* = .064) (Table 1). Posters exhibited higher levels of perceived stress (*F*(2, 215) = 6.778, *p* = .001) and anxiety symptoms (*F*(2, 207) = 6.406, *p* = .002) compared to lurkers and non-users at intake (Table 2). There were no significant differences between non-users, lurkers and posters in education level, household income, ethnicity, immigrant status, type of infertility diagnosis, or whether or not they achieved a pregnancy throughout the study period (Table 1).

**Table 1.** Differences in socio-demographic and fertility-related characteristics between posters, lurkers and non-users of the *Connect* forum.

	Full sample		Non-users		Lurkers		Posters			
	n	Valid %	n	Valid %	n	Valid %	n	Valid %	X^2^	*p*
Sex	220	–	114	–	66	–	40	–	14.682	.**001**
Male	50	22.73	37	32.46	11	16.67	2	5.00		
Female	170	7727	77	67.54	55	83.33	38	95.00		
Education	218	–	112	–	66	–	40	–	1.915	.927
High school or less	16	7.34	7	6.25	7	10.61	2	5.00		
CEGEP, vocational	39	17.89	21	18.75	11	16.67	7	17.50		
University degree	72	33.03	36	32.14	21	31.82	15	37.50		
Graduate degree	91	41.74	48	42.86	27	40.91	16	40.00		
Missing	2	–	2	–	0	–	0	–		
Household income ($)	215	–	110	–	66	–	39	–	1.610	.952
Less than 80,000	62	28.84	31	28.18	21	31.82	10	25.64		
80,000–119,999	64	29.77	31	28.18	21	31.82	12	30.77		
120,000–159,999	40	18.60	20	18.18	12	18.18	8	20.51		
160,000 and over	49	22.79	28	25.45	12	18.18	9	23.08		
Missing	5	–	4	–	0	–	1	–		
Ethnicity	217	–	111	–	66	–	40	–	.574	.751
White	131	60.37	67	60.36	38	57.58	26	65.00		
Non-white	86	39.63	44	39.64	28	42.42	14	35.00		
Missing	3	–	3	–	0	–	0	–		
Immigration status	215	–	109	–	66	–	40	–	1.754	.416
Immigrant	88	40.93	49	44.95	23	34.85	16	40.00		
Born in Canada	127	59.07	60	55.05	43	65.15	24	60.00		
Missing	5	–	5	–	0	–	0	–		
Parity	217	–	112	–	65	–	40	–	5.493	.**064**
No children	174	80.18	83	74.11	57	87.69	34	85.00		
1 child or more	43	19.82	29	25.89	8	12.31	6	15.00		
Missing	3	–	2	–	1	–	0	–		
Prior psychological counselling	218	–	112	–	66	–	40	–	9.222	.**010**
Yes	35	16.06	11	9.82	12	18.18	12	30.00		
No	183	83.94	101	90.18	54	81.82	28	70.00		
Missing	2	–	2	–	0	–	0			
Types of diagnoses	219	–	113	–	66	–	40	–	3.599	.891
Female-factor only	71	32.42	34	30.09	25	37.88	12	31.00		
Male-factor only	53	24.20	28	24.78	17	25.76	8	20.00		
Mixed (male and female factor)	30	13.70	16	14.16	7	10.61	7	17.50		
Unexplained/In testing	65	29.68	35	30.97	17	25.76	13	32.50		
Missing	1	–	1	–	0	–	0	–		
Time in fertility treatment	212	–	109	–	63	–	40	–	17.353	.**008**
Less than 5 months	56	26.42	37	33.94	16	25.40	3	7.50		
5–12 months	56	26.42	26	23.85	15	23.81	15	37.50		
13–30 months	51	24.06	20	18.35	15	23.81	16	40.00		
31 months or more	49	23.11	26	23.85	17	26.98	6	15.00		
Missing	8	–	5	–	3	–	0	–		
Achieved pregnancy during study	172	–	82	–	51	–	39	–	.100	.951
No	129	75.00	61	74.39	38	74.51	30	76.92		
Yes	43	25.00	21	25.61	13	25.49	9	23.08		
Missing	48	–	32	–	15	–	1	–		

Abbreviations: n = number of cases; X^2^ = Chi-square test; *p* = significance value (2-sided test).

**Table 2.** Differences in psychological outcomes at intake between posters, lurkers and non-users of the *Connect* forum.

			Non-user		Lurker		Poster		ANOVA	
	n	Missing	*M*	*SD*	*M*	*SD*	*M*	*SD*	F	*p*
Anxiety symptoms (GAD-7)	210	10	7.31	5.85	5.95	4.55	10.00^a^	5.82	6.406	.**002**
Perceived Stress (PSS-4)	218	2	6.59	2.94	6.59	3.13	8.63^b^	3.54	6.778	.**001**

a = Bonferroni post-hoc shows that posters had significantly higher GAD-7 scores than lurkers (*p* = 0.001, mean difference = 4.047, SD = 1.132) and non-users (*p* = 0.032, mean difference = 2.688, SD = 1.043).

b =  Bonferroni post-hoc shows that posters had significantly higher PSS-4 scores than lurkers (*p* = 0.004, mean difference = 2.041, SD = 633) and non-users (*p* = 0.002, mean difference = 2.044, SD = .582).

*Abbreviations.* GAD-7 = Generalized Anxiety Disorder 7-item scale; PSS-4 = Perceived Stress Scale 4; n = number of cases; M = mean; SD = standard deviation; *p* = significance value (2-sided test).

Model 1 (Table 3) and Model 2 (Table 4) present the results of the multinomial logistic regressions. Model 1 was statistically significant (X^2^ (10)  =  45.501, *p* < 0.001). The adjusted R^2^ (Cox and Snell) indicated that 20.3% of the variance in *Connect* use was explained by the model. Having more anxiety symptoms (GAD-7) before using the app was significantly associated with being a poster rather than a lurker (*p* = 0.002) or non-user (*p* = 0.087), after controlling for time in treatment, sex, parity and prior psychological counselling. Being female, not having children, and prior psychological counselling remained significant predictors of posting on *Connect* after controlling for covariates in the logistic regression model (see Table 3).

**Table 3.** Regression analysis summary of whether anxiety symptoms at intake predict use of the *Connect* forum.

Model 1	*B*	*SE*	Wald	df	Exp(B)	95% CI	*p*
**Non-user vs. Poster**							
Anxiety symptoms (GAD-7)	−.062	.036	2.927	1	.940	[.875, 1.009]	.**087**
Time in treatment	−.236	.201	1.373	1	.790	[.532, 1.172]	.241
Being male	2.044	.779	6.893	1	7.724	[1.679, 35.531]	.**009**
No children	−1.502	.614	5.978	1	.223	[.067, .742]	.**014**
No prior psychological counselling	1.065	.521	4.170	1	2.900	[1.044, 8.056]	.**041**
**Lurker vs. Poster**							
Anxiety symptoms (GAD-7)	−.126	.041	9.380	1	.882	[.814, .956]	.**002**
Time in treatment	.017	.212	.007	1	1.017	[.671, 1.543]	.935
Being male	.894	.831	1.157	1	2.444	[.480, 12.452]	.282
No children	−.086	.703	.015	1	.918	[.232, 3.639]	.903
No prior psychological counselling	.402	.511	.620	1	1.495	[.549, 4.072]	.431

Note: Posters were used as the reference group.

Abbreviations: GAD-7 = Generalized Anxiety Disorder 7-item scale; SE = standard error; df = degrees of freedom; CI  =  Confidence interval for Exp(B); *p* = significance value (2-sided test).

**Table 4.** Regression analysis summary of whether perceived stress levels at intake predict use of the *Connect* forum.

Model 2	*B*	*SE*	Wald	df	Exp(B)	95% CI	*p*
**Non-user vs. Poster**							
Stress (PSS-4)	−.159	.069	5.275	1	.853	[.745, .977]	.**022**
Time in treatment	−.171	.197	.748	1	.843	[.573, 1.241]	.387
Being male	2.047	.779	6.901	1	7.743	[1.681, 35.659]	.**009**
No children	−.889	.543	2.684	1	.411	[.142, 1.191]	.101
No prior psychological counselling	1.106	.518	4.569	1	3.023	[1.096, 8.337]	.**033**
**Lurker vs. Poster**							
Stress (PSS-4)	−.203	.073	7.772	1	.816	[.707, .941]	.**005**
Time in treatment	.081	.208	.151	1	1.084	[.721, 1.631]	.697
Being male	.924	.826	1.253	1	1.906	[.345, 10.539]	.460
No children	.361	.640	.319	1	1.435	[.410, 5.029]	.572
No prior psychological counselling	.830	.578	2.063	1	2.294	[.739, 7.122]	.151

Note: Posters were used as the reference group.

Abbreviations: PSS-4 = Perceived Stress Scale 4; SE = standard error; df = degrees of freedom; CI  =  Confidence interval for Exp(B); *p* = significance value (2-sided test).

Model 2 was statistically significant (X^2^ (14)  =  42.527, *p* < 0.001). The adjusted R^2^ (Cox and Snell) indicated that 18.4% of the variance in *Connect* use was explained by the model. Having higher levels of perceived stress (PSS-4) before using the app was significantly associated with being a poster rather than a lurker (*p* = 0.005) or non-user (*p* = 0.022), after controlling for time in treatment, sex, parity, and prior psychological counselling. Being female and prior psychological counselling remained significant predictors of being a poster after controlling for covariates in the logistic regression model (see Table 4).

Non-users and lurkers had no significant changes in PSS-4 or GAD-7 scores from intake to follow-up questionnaires after app-use. However, posters scored significantly lower on both the PSS-4 and GAD-7 at follow-up compared to intake, meaning that they had lower levels of perceived stress and anxiety symptoms after using the app (Table 5). The effect of *Connect* use on changes in PSS-4 and GAD-7 scores from intake to follow-up were further analyzed using repeated-measures ANCOVAs to control for the covariates sex, parity, time in treatment, prior psychological counselling, and whether or not the participant achieved pregnancy during the study period (Table 6). The interaction of time (from intake to follow-up) and *Connect* use was marginally associated with anxiety symptoms (GAD-7) with a *p*-value of 0.083, however it was not significantly associated with perceived stress (PSS-4).

**Table 5.** Changes in perceived stress and anxiety symptoms from intake to follow-up based on *Connect* use.

	Posters				Lurkers				Non-users			
	*M*	*SD*	df	*p*	*M*	*SD*	df	*p*	*M*	*SD*	df	*p*
Perceived stress (PSS-4)												
Intake	8.49	3.48	36	.**059**	6.33	3.28	50	.279	6.63	2.85	79	.901
Follow-up	7.51	3.12			5.98	2.76			6.59	3.08		
Anxiety symptoms (GAD-7)												
Intake	9.51	5.57	34	.**004**	5.46	4.43	47	.721	6.79	5.49	74	.289
Follow-up	6.89	5.18			5.29	4.79			6.20	5.09		

Note. Significant *p* indicates a significant score change between time-points (2-sided test). Direction of change is observable with the mean.

Abbreviations*:* GAD-7 = Generalized Anxiety Disorder 7-item scale; PSS-4 = Perceived Stress Scale 4; M = mean; SD = standard deviation; df = degrees of freedom.

**Table 6.** Comparing participants’ changes in mean scores from intake to follow-up (repeated-measures ANCOVAs).

Effect	MS	df	F	*p*	Partial Eta Square
Anxiety symptoms (GAD-7)					
Time (Intake to follow-up)	19.531	1	2.026	.157	.014
Time x Connect use	24.383	2	2.529	**.083**	.034
Time x Successful pregnancy	18.017	1	1.869	.174	.013
Error	9.641	143			
Perceived stress (PSS-4)					
Time (Intake to follow-up)	1.068	1	.297	.587	.002
Time x Connect use	6.935	2	1.929	.149	.025
Time x Successful pregnancy	12.315	1	3.426	.**066**	.022
Error	3.595	153			

Note: The repeated-measures ANCOVAs controlled for covariates that were significant in bivariate analyses: sex, parity, previous psychological counselling, and time in treatment.

Abbreviations*:* GAD-7 = Generalized Anxiety Disorder 7-item scale; PSS-4 = Perceived Stress Scale 4; MS = mean square; df = degrees of freedom; F = F-statistic from repeated-measures ANCOVAs; *p* = significance value.

Of the 106 participants who used *Connect*, 42 completed the PSEI. Posters (M = 51.93, SD = 9.09) scored significantly higher than lurkers (M = 44.57, SD = 8.96; *t*(40) = −2.484, *p* = .017), suggesting that posters reported a more positive experience with the peer supporters and the *Connect* forum overall than lurkers.

### Experiences using the *Connect* forum

In total, 40 participants posted on the *Connect* forum, making a total of 244 posts. After the eight-week study period, 50 participants left qualitative feedback about their *Connect* use and peer supporter interactions, 28 of whom were posters, and 22 lurkers. The participants with qualitative data did not significantly differ from the total sample of *Connect* users in demographic characteristics and psychological outcomes; however, they were more likely to be posters (X^2^ (1, N = 106)  =  19.955, *p* = .000) and those with higher PSEI scores (F(1, 40) = 5.335, *p* = .026). Thirty-eight of these 50 (76%) participants expressed a positive overall experience with the *Connect* forum. A number of themes emerged through thematic analysis of the open-ended questions on the surveys and the qualitative interviews.

**1. Reduced isolation: ‘I felt less alone knowing I could contribute to the forum.’** The most common theme that arose was how having access to the peer support forum reduced feelings of isolation. For some, simply ‘knowing there are real people going through the struggles of infertility’ (#5, female) was what they enjoyed about *Connect*. Being able to see what others were going through made participants feel validated: ‘many doubts were also my own’ (#3, female).

Other participants appreciated the opportunity ‘to be able to talk and connect with people going through the same issues’ (#46, female). One participant said: ‘to see that you aren’t alone when going through these fertility problems by getting and or giving advice. Talking to someone who doesn’t have problems isn’t the same – they don’t understand’ (#26, female).

Some participants preferred lurking rather than posting: ‘I've been a sort of ‘invisible stalker.’ I go on these forums and I read all the symptoms … I try to see what other women have’ explained one participant (#18, female). Having the opportunity to lurk was beneficial for those who felt shy to post themselves: ‘I was timid to share my thoughts. But I read others’ comments and experiences and healed through them. It was nice to hear the male's struggles as often enough we think it's just the women who are affected’ (#5, female).

Finally, some participants expressed that *Connect* made them feel like they were part of a community: ‘We would look at [the posts] just to see what other people are going through. You know, it would bring a sense of community to the issue’ (#20, male). One participant said that they appreciated ‘being able to talk with others and try to help by giving my own experiences’ (#39, female). The ability to help others was a rewarding experience, and one participant explained that to contribute, they would seek out message threads to provide support and advice. When asked about what it was like to provide support to others, this participant explained that it brings ‘this sense of, you know, ‘we’re all in this together. We’re all struggling’’ (#18, female). Being able to help others was a way to reduce isolation and build a community.

**2. Management and reduction of stress.** Many participants expressed that the *Connect* forum provided comfort and support, helped users to reduce their stress and allowed them to learn how others manage stress: ‘although I didn’t use it as my own soundboard, I sought comfort in reading the support given to others’ (#5, female).

Another participant used *Connect* to help improve her mood when she was feeling down: ‘I was happy to go [on *Connect*] when I didn't feel well and was questioning myself. When you don't feel well, you don't stay in that zone. You try to get out of it’ (#34, female).

Participants also found that the ability to express themselves among people that understood what they were going through helped them manage and reduce stress. ‘I liked the peer support very much, it helped me calm down and feel like I have someone I can vent to’ explained one participant (#18, female). The forum was an important outlet for this participant: ‘To have a safe space where I can go nuts for a little bit and then calm down. That's what I really liked.’ (#18, female).

Finally, participants used the forum to read about how others cope with the emotional and physical tolls of infertility. One participant explained how *Connect* offered him and his wife a tangible way to cope with their anxiety:

There was a chat that was about the two-week waiting time and we were just beginning the two-week waiting time so that really came at the good moment. They were giving tips on how to deal with the stress and the anxiety during the two-week waiting time. And one woman was just saying, ‘I usually just do puzzles … To kill off the time and, you know, not to think about stuff.’ And I was like, ‘my wife loves puzzles, so maybe we should do that.’ And I went to the second hand store and I got four of them and I came back that night with four of them and my wife was like, ‘what are you doing?’ ‘I think it’s gonna be good for you … we can work on these together’ … And she was like, ‘that’s a great idea.’ So, it came in really handy to me because it was what I needed at that time (#40, male).

Reading about others’ experiences was a validating experience and helped participants cope with stress: ‘What I benefitted from was the opportunity to read how other people were coping with their situations and to be able to post my own thoughts/feelings’ (#50, female).

**3. The benefits of trained peer supporters monitoring the forum.** Many participants talked about the benefits of having trained peer supporters who monitored the forum: ‘I would go online and, you know, vocalize my feelings and someone would get back – a peer supporter. And that was very helpful’ (#18, female). Peers ensured that the forum remained respectful and on topic and that no post was left unanswered: ‘I like that there are Infotility Peer Support people who participate, because it helps keep the topic on track and hearing from them is reassuring’ said one participant (#7, female). Another said they ‘liked the forum questions because they were kept respectful and the peer supporters were available’ (#42, female).

A few participants expressed that the peer supporters provided them with a type of support that they were unable to find at clinics and with doctors: ‘The clinic gives you almost ZERO empathy, something that I found in this app’ (#40, male).

**4. Valuable and diverse information.** Multiple participants expressed that they found *Connect* to be a valuable source of information on a variety of fertility topics and that they received ‘useful answers from people with actual experience’ (#2, female). ‘I enjoyed the fact that I can go on there and ask questions,’ said one participant who felt that other users were ‘not only sympathetic, but they also have a good idea what the answer is to my question specifically’ (#20, male).

Many appreciated reading about a range of fertility topics, including ones that they had never thought about before: ‘I was blown away by the dilemmas people were facing ahead of me … I found that helpful because those would have never been things I would have even considered’ (#30, female). Being exposed to a range of topics and experiences was a beneficial source of information: ‘You get to find out new things that you didn’t consider before … [the *Connect* forum] was a good place to go and find some information’ (#40, male).

One participant was grateful to have *Connect* when starting a new phase of treatment: ‘Now that I’m gonna start IVF I might go on again and check what people are saying, because now I’m at a different stage, and I now have a different set of questions’ (#30, female).

Another participant appreciated the detail and depth of the messages that were posted: ‘It was interesting anyway. They weren’t necessarily short answers. There are people who really elaborate on the subject, which I thought was really cool’ (#22, female).

*Connect* was a positive and non-judgemental environment in which participants could learn new information: ‘I liked the discussion board. I was able to express myself without judgement and get answers to questions I had’ (#45, female).

**5. Confidentiality: ‘I felt it was safe.’** Participants appreciated the confidential nature of *Connect* and expressed that it encouraged them to use it. ‘Fertility problems are not something you go around and talk freely with all your friends about’ said one participant (#40, male) when describing why he appreciated the opportunity to use *Connect*. Another participant expressed that the private nature of *Connect* fostered a safe space: ‘I felt it was safe. I liked that they asked you to have a username that was not personal’ (#30, female).

**6. Limitations of the *Connect* forum.** While the majority of participants had a positive experience on the forum, there were a number of limitations. Many participants expressed that they would have benefitted from the forum being more populated and having more consistent interaction. ‘There are currently too few users for it to be truly helpful in terms of peer support. Postings receive a few responses,’ explained one user (#38, female). Since the app was only available to participants enrolled in the research study and not to the general public, there was a limited number of participants on the forum.

Some participants also felt that responses from the peer supporters were too broad, and ‘not equivalent to conversing directly with someone who has experienced fertility issues’ (#33, female). Peer supporters were trained not to provide medical information, and not to mention specific brands, types of medications, or clinic names. Some participants felt this was insufficient as they wanted to be able to communicate with more details and directly with medical professionals.

Participants also wanted to be able to receive notifications when new comments were posted and to send private messages to other participants (this was prohibited as it meant peers would not be able to monitor the messages).

Finally, some participants felt that the topics discussed on *Connect* were not relevant to them. One man noted that there was a lack of discussion from men: ‘I didn’t find too much peer support from guys; I think it is mainly women replying there … I think it would have been more interesting to get some feedback from other men that have been going through this’ (#40, male). Some participants also had difficulties finding others in similar circumstances: ‘I guess everyone’s fertility issues are a little bit different … It’s very hard to find something that relates to your situation that you're going through’ (#31, male). As a potential solution to this limitation, one participant suggested that ‘it would be helpful to have separate “chat rooms” for patients at the same stage of treatment’ (#36, female).

## Discussion

The purpose of this study was to explore the use of an online peer support forum in a sample of people experiencing fertility difficulties. By adopting a mixed-methods approach, we attempted to understand why certain people used the forum while others did not, how users described their experiences on the forum, and how different levels of engagement were related to levels of perceived stress and anxiety symptoms. Our study is the first to compare posters, lurkers, as well as non-users of fertility-related peer support forums on a number of psychological and socio-demographic measures. The results demonstrate that the *Connect* forum was of significant interest to fertility patients, particularly those experiencing greater psychological distress. Both perceived stress and anxiety symptoms were associated with being more active on the forum. Importantly, posters had reduced levels of anxiety after eight weeks of app use, independent of whether or not they achieved a pregnancy during the study period and controlling for other covariates significant in bivariate analyses. Users described their experiences as predominantly positive, highlighting how the forum reduced their feelings of isolation, helped them manage the stress of infertility, exposed them to valuable and diverse information, and made them feel safe and part of a community. The peer supporters contributed to participants’ positive experiences by being respectful, available, non-judgemental, and keeping posts on topic. The results of this study highlight a need within the infertility community for peer support forums which are confidential and supportive.

Many participants expressed that the *Connect* forum provided them with new ways to manage their stress by offering a safe environment to vent and reduce their isolation. Quantitative findings support these qualitative data. While the study design does not permit us to conclude that there were direct effects of using the *Connect* forum on participants’ psychological distress levels, results show that posters reported lower levels of anxiety and perceived stress at follow-up compared to intake. In contrast, lurkers and non-users showed no significant changes. Those with heightened distress may have posted in search of emotional support and connection with others going through similar experiences (Malik & Coulson, 2011; van Uden-Kraan, Drossaert, Taal, Shaw, et al., 2008). *Connect* users appreciated the opportunity to share their own experiences and communicate with similar others, which may have helped them feel supported and reappraise their situation to reduce their distress.

Similarly, our results show that those who had sought psychological counselling in the past were more likely to post on *Connect*. This aligns with literature suggesting that forums are often used as supplementary forms of emotional and appraisal support in addition to traditional forms of counselling (Kummervold et al., 2002). The association between prior psychological counselling and forum use also suggests that some patients may be more proactive in locating and making use of support. Despite the association between prior psychological counselling and using *Connect* there were many users who had never sought psychological counselling who still benefitted from using the forum based on their qualitative feedback.

We also found that posters and lurkers had been in fertility treatment longer than non-users. This suggests that fertility patients who were just starting their fertility journey may not have felt the need to communicate and seek support from others on the forum, and may have been satisfied with just using the informational side of the app. On the other hand, those participants who had been visiting fertility specialists for longer periods of time may have been more likely to have experienced treatment failure in the past, and more inclined to seek out support on the *Connect* forum to communicate with those who may understand what they are going through. Furthermore, it is possible that those who had been in fertility treatment longer had learned how to better manage the stress and anxiety that can arise from fertility treatment, and as a result felt the desire to share their experiences and offer support to others on the forum. This hypothesis is supported by the qualitative results, which highlighted how some posters sought out discussion posts in order to provide support and advice because they felt that the ability to do so was rewarding.

It is also interesting to note that those who posted rather than lurked rated their experiences on *Connect* more positively. This suggests that being actively engaged in conversation and connection with others may be more beneficial for users, which is in line with previous research (Malik & Coulson, 2011; Preece et al., 2004). Compared to posters, lurkers may have had a less positive experience because they did not find the conversations on the forum to be personally relevant. The qualitative findings show that some participants felt that the responses from peer supporters were too broad and they did not feel the discussions from other users were relevant to their own situation. One participant suggested having different chat rooms tailored to specific diagnoses or infertility experiences as a potential solution to this limitation. Participants might not have been comfortable posting or responding to others if the topics were not relevant to their own experiences.

Men in particular may have felt as though the topics discussed were not relevant to their experiences. This might help to explain why men were much more likely to be lurkers rather than posters; as more and more women posted on the forum and conversed with each other, men might have felt increasingly isolated from the discussions. One of the few men who actively posted on the forum was disappointed by the lack of male-specific topics and interaction among men. Despite the fact that there were male peer supporters who attempted to engage men, women were still significantly more likely to post on the forum.

Previous research has also found that men are less likely to use forums than women (Grande, Myers, & Sutton, 2006; Steginga et al., 2008), and in particular, that forums geared towards men are more likely to be information-focused, whereas those for women are more likely to focus on emotional support (Mo, Malik, & Coulson, 2009). *Connect* was geared towards emotional support, rather than the provision of information, and participants were encouraged to speak to their doctors about medical advice and treatments rather than using the forum for these purposes. This may help to explain why men were less likely to use the forum.

Finally, even though participants were allowed to continue using the app after providing feedback at the end of the eight-week study period, it is possible that some participants, namely lurkers, did not become fully familiarized or comfortable with using the *Connect* forum before being asked to provide feedback. Previous literature has found that higher levels of familiarity with a forum develops through involvement over time and eventually leads a lurker to become a poster (Rafaeli, Ravid, & Soroka, 2004). Nonetheless, the qualitative data highlight that many lurkers still benefitted from and enjoyed the forum.

Online support resources should take into consideration how infertility experiences differ based on sex, diagnosis, or length of time trying to conceive. Making forums relevant and welcoming to those with differing experiences may encourage involvement and improve experiences.

### Strengths and limitations

Mixed-methods research allows for a comprehensive understanding of the research questions and greater validity of findings through corroboration of quantitative and qualitative data (Doyle, Brady, & Byrne, 2009). Understanding who was more likely to be engaged with *Connect* could not have been adequately answered with only qualitative data and understanding peoples’ experiences using *Connect* could not have been adequately answered with the quantitative data. Comparing our qualitative and quantitative data allows for a nuanced understanding of who benefits from the use of forums, and the nature of the benefits.

Despite these strengths, there are certain limitations to the present study. Participants were recruited in fertility clinic waiting rooms; as a result, the current study only analyzed data from those who were pursuing fertility treatment. This limited the sample, as it excluded people with fertility concerns who may use forums but who are not pursuing medical treatment. In addition, participants were divided into non-users, lurkers or posters in order to measure use of the *Connect* forum. This variable does not take into consideration varying levels of engagement within these categories, such as posters who only posted once versus multiple times. This reduces the complexity of the findings. Furthermore, while our results did take into consideration the length of time in treatment, we did not control for fertility treatment history which may have affected satisfaction with and use of the forum. We also were unable to examine the reasons for which non-users decided not to use the forum; our qualitative data analysis was focused only on *Connect* users, and we did not ask non-users about why they decided not to use the forum in the qualitative interviews. Finally, the current study is limited by the relatively small sample size and the drop out rate. This may have reduced the statistical power of the quantitative results, particularly the multivariate tests that were run, and limits the external validity of the findings.

## Conclusion

The results of this study highlight a need within the infertility community for online peer support forums. The current study expands on existing literature by adopting a mixed-methods approach to understand who uses forums and who benefits from them. To the best of our knowledge, this study is the first to compare posters, lurkers, and non-users of fertility-related peer support forums on a number of psychological and socio-demographic measures. Those with heightened psychological distress sought out involvement in the forum when presented with such a resource. Participants who used the forum appreciated the opportunity to share their own experiences and communicate with others in similar situations. It made them feel less alone, allowed them to learn new ways to appraise and manage their stress, and provided social comparisons. The results highlight the many important benefits of monitored peer support forums for fertility patients, as well as certain improvements that could be made to encourage use. Having trained peer supporters monitor the forum kept discussions respectful and on-topic. Clinics and health care providers may consider providing fertility patients with a list of safe and monitored online forums, which can offer much needed support. Future research should expand upon this study to measure how forums such as *Connect* can serve to reduce psychological distress and improve quality of life among those undergoing fertility treatment through randomized controlled trials. In addition, future research should design online forums that can be tailored to specific fertility-related experiences, to encourage use and improve experiences.

## Data Availability

The data that support the findings of this study are available upon reasonable request from the corresponding author, P.Z. The data are not publicly available to protect the privacy of individuals who participated in the study.

## Appendix 1. Peer Support Evaluation Inventory (PSEI)

Instructions for participants: ‘For the following questions, please consider your experiences with all of the peer support volunteers with whom you had contact. Whether you posted one or more times, try to answer based on an average of the encounters you had. If you did not interact with the peers, please skip this questionnaire (hit the submit button without filling any answers).’

Answer options for each question:

0 = Strongly disagree; 1 = Somewhat disagree; 2 = Somewhat agree; 3 = Strongly agree.

Part 1: In general, peers:

1. Provided me with practical information.
2. Provided valuable personal information that helped me think about my own situation.
3. Responded to posts in a timely fashion.

Part 2: In general:

1. I could share important experiences with peers.
2. I felt peers had empathy for my situation.
3. Peers were an important source of support for me.
4. The messages that peers wrote were too long.
5. The messages that peers wrote were too short.
6. Peers minimized my problems.

Part 3: Over the past 2 months, peers helped me feel:

1. I have something in common with other people going through my fertility treatment.
2. Less isolated from others
3. More knowledgeable about my situation.

Part 4:

1. Peers met my expectations.
2. I would recommend this type of support to a friend with fertility issues.
3. I am satisfied with my peer support service.
4. Receiving support from peers via the app was convenient for me.
5. Is there anything else you would like to tell us about your peer support experience? (Open-ended question).
